# Supplementary material for: Re-entrant DNA gels
Source: Nat Commun. 2016 Oct 21;7:13191. doi: 10.1038/ncomms13191 (PMC5078732; doi:10.1038/ncomms13191)
Supplement: Supplementary Information — Supplementary Figures 1 - 8 and Supplementary Discussion [file ncomms13191-s1.pdf]

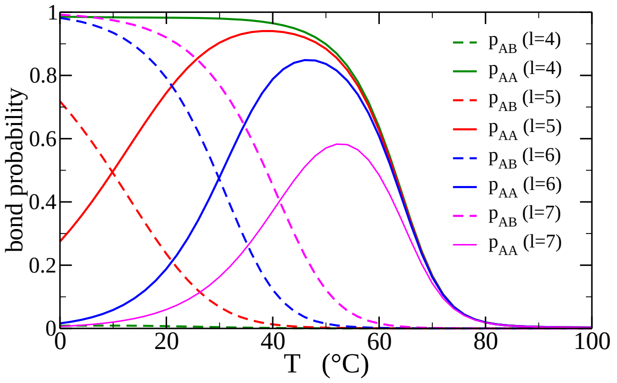

Supplementary Figure 1: **Bonding probabilities.** Probability of AA binding (p_{AA}) and AB binding (p_{AB}) as a function of T for different lengths l of the competitor sequences. Here the concentration of each strand is $c = 8.52 \cdot 10^{-4}$ M and [NaCl]=0.13 M.

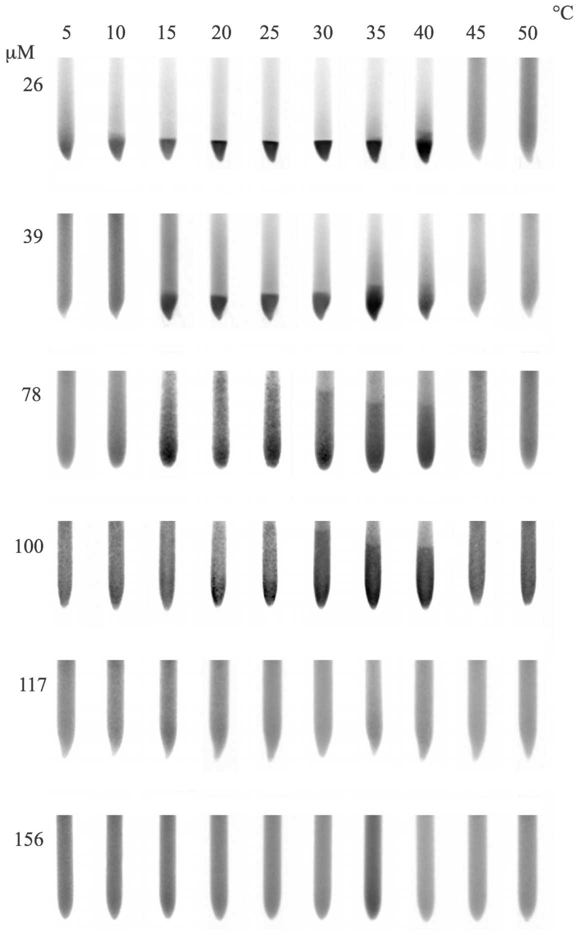

Supplementary Figure 2: **Temperature-dependence of the phase-separation meniscus for samples at increasing nano-star concentration (26, 39, 78, 100, 117 and 156 μM).** Samples marked with EtBr were inserted in home-made microcapillary cells and centrifuged overnight (at 4400 rpm) at specific target temperatures to speed up the macroscopic phase separation. After centrifugation, each capillary was promptly photographed with a BIO-RAD ChemiDoc (at the same temperature at which it was centrifuged) to evaluate the position of the meniscus.

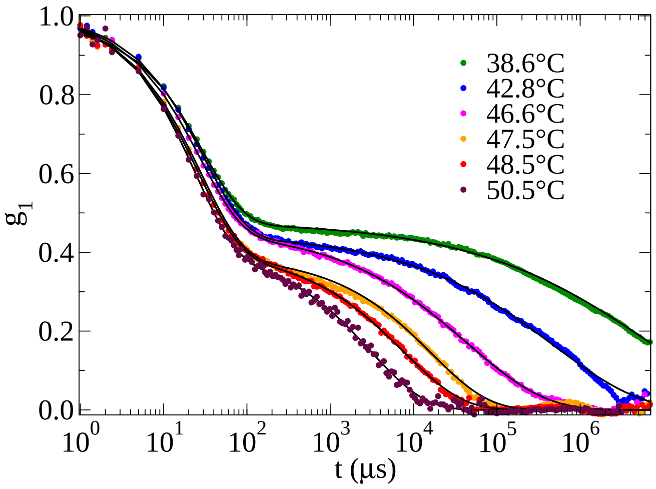

Supplementary Figure 3: **Dynamic light scattering correlation functions for different temperatures at $c = 213$ μM fitted to the sum of two stretched exponentials.** The black solid lines indicate the fitting function.

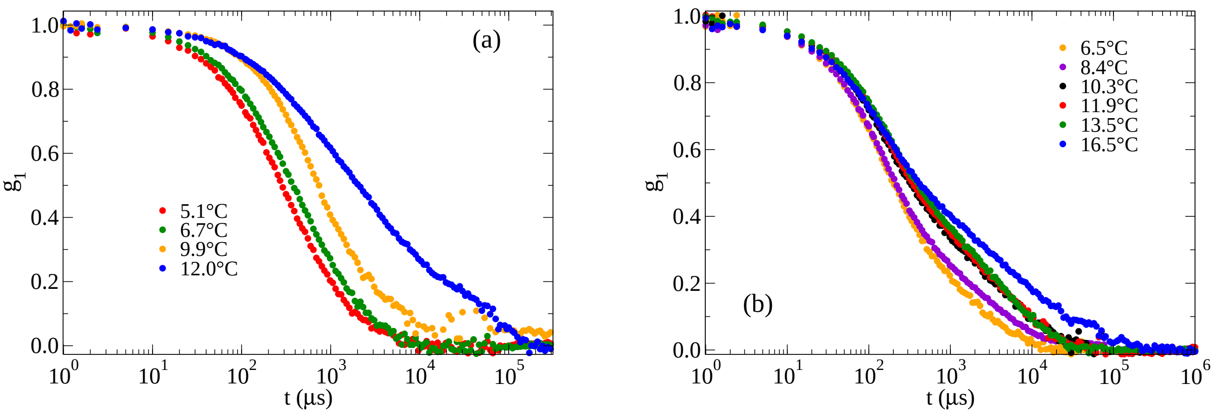

Supplementary Figure 4: **Dynamic light scattering correlation functions at low concentration.** (a) The region of gel breaking (low-temperatures), where the decay time of the correlation functions progressively decreases, for the sample at $c_1 = 39$ μM; (b) The region of gel breaking (low-temperatures) for the sample at $c_2 = 78$ μM.

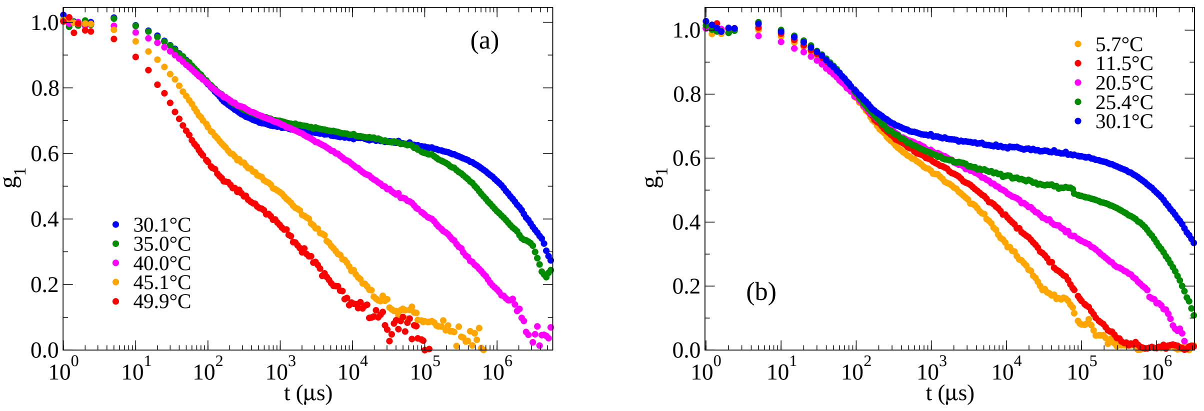

Supplementary Figure 5: **Dynamic light scattering correlation functions for different temperatures at $c = 117\mu$M.** (a) The region of gel formation where the decay time of the correlation functions progressively increases going beyond what can be experimentally measured ($\approx$ 10 s) when $T \approx 30^{\circ}C$. (b) The region of gel breaking, where the decay time of the correlation functions progressively decreases, indicating the melting of the network and the formation of AB_4 structures.

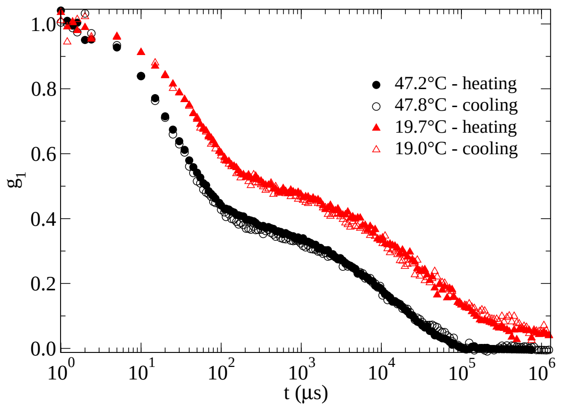

Supplementary Figure 6: **Evidence of reversible behavior** Dynamic light scattering correlation functions for a sample at concentration 15 mg/ml (213 μM) at two different Ts, in the pre-gelling and in the post-melting region on a cooling path and in the pre-gelling and in the post-melting region on heating, following a few hour stay at 5°C. At corresponding T, the correlation function is comparable, providing evidence of full reversibility.

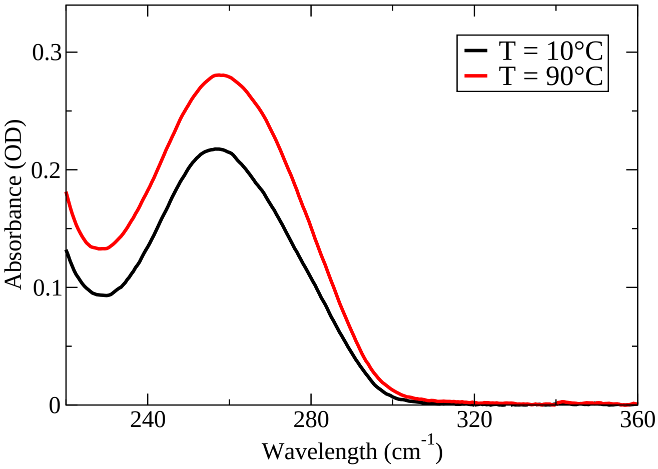

Supplementary Figure 7: **Comparison between the UV absorption spectra at 90°C and 10°C.** The height of the peaks at 260 nm evidences a marked hyper-chromism (the absorbance of single-stranded DNA, 90°C, is higher than the one of double-stranded DNA, 10°C) which allow for the investigation of the melting process.

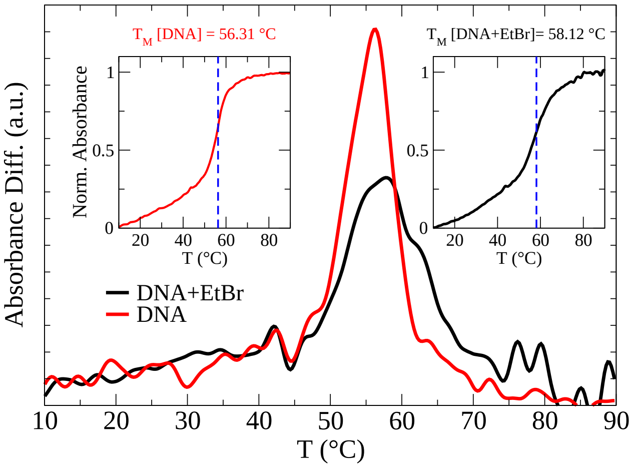

Supplementary Figure 8: **Effect of EtBr on the melting temperature of the system.** Comparison between the first derivatives of the UV absorbance curves in absence (red) and in presence (black) of EtBr. The position of the maximum is associated to the melting T. The insets show the measured absorbance curves (blue dashed lines indicates the melting (maximum slope) T).

Supplementary Discussion

Supplementary discussion: Sequence design

We request an all-DNA system that is able to assume four different states (corresponding to the ones indicated in Fig. 1(a-d) of the main text) in a range of temperatures (0 - 90)°C. These states are separated by three characteristic temperatures, $T_{\rm high}$, $T_{\rm network}$ and T_{AB_4}. The system must be

- at very high T ($T > T_{\rm high}$), a solution of single strands (Fig. 1-(a));
- at high T ($T_{\rm network} < T < T_{\rm high}$), a solution of (almost) non-interacting four-armed DNA nano-stars (Fig. 1-(b));
- at intermediate T ($T_{AB_4} < T < T_{\rm network}$), a highly connected network of DNA nano-stars (Fig. 1-(c));
- at low T ($T < T_{AB_4}$), a solution of (almost) non-interacting DNA nano-stars with sticky arms saturated by competing sequences (Fig. 1-(d)).

The highest of these temperatures, $T_{\rm high}$, corresponds to the hybridization temperature of long (i.e. > 20) stretches of DNA and it is fixed almost entirely by the ionic strength, being very weakly dependent on either number of base pairs or DNA concentration (this is different from the case of short DNA strands, for which the melting temperature is steeply dependent on both the the number of base pairs and the concentration).

At fixed nano-star concentration, $T_{\rm network}$ is set by the number n_{AA} of base pairs that two tetramers form when binding to each other. We request $T_{\rm network}$ to be as high as possible, but still well separated from $T_{\rm high}$. For this reason, we choose sticky ends that form 8 base pairs. A larger number of base pairs would shift $T_{\rm network}$ too close to $T_{\rm high}$. In addition, since the AA sequence needs to be self-complementary to allow for AA bonding, a longer sequence would favour the formation of hairpin structures (detrimental for the gel formation). T_{AB_4} depends on the number of binding bases of the competitor (the B particle) $n_{\rm AB}$ and on their molar concentration $c_{competitor}$: in principle, it is possible to use an excess of competitors, e.g. a concentration of competitor strands much larger than the molar concentration of nano-stars sticky ends c_A, but this would increase T_{AB_4}. Absence of AA bonds at low T imposes $c_{competitor} \geq c_A$. We also require T_{AB_4} to be significantly lower than $T_{\rm network}$.

We used NUPACKto design the sequences. There are only $(8/2)^4 = 256$ palindromic sequences of length eight, so we can test all of them in a variety of situations. To design the competing sequence, we take into account that they need to displace, for a large part if not all, the AA bonds. Hence, if the same sequence needs to displace more than four bases (out of the eight in the AA bond), it will need to be also at least partially self-complementary. This poses a threat, since the self-complementary parts of the B strands can associate forming unwanted BB bonds. For the reasons above, it is inconvenient to design an all-DNA reversible gel with a single B-particle. The solution we propose is to use two different competing sequences per arm, each one replacing three of the base pairs of the AA bond, for a total of 6 replaced base pairs. To favour the switching process we add at the two extremes of the 8-base sticky sequence additional (non-self-complementary) bases which can act as anchoring points for the competing sequences to initiate the swapping process. These extra binding bases also stabilize the AB bonds, increasing the probability to form AB bonds with respect to AA ones. Further, the use of a higher number of shorter sequences allows to increase the entropy cost of the AB bonds. This allows us to make the transition sharper, and thus interfere less with the network at intermediate temperatures.

To decide the optimal number of toehold bases, we study the T dependence of p_{AA} and p_{AB} assuming a stoichiometric concentration of sticky ends and competing strands as a function of the total length of the competitor. The results are shown in Supplementary Fig. 1. The optimal value (the one for which p_{AA} is largest at intermediate T and p_{AB} approaches one at low T) is found for a total length of the competitor sequence equal to six. This is the value chosen in the experimental study.

Supplementary discussion: Phase diagram

By following the protocol described in Materials and Methods, it was possible to accurately monitor the temperature dependence of the meniscus in the phase-separating samples at different nano-star concentrations: 26, 39, 78, 100, 117, 156 μM. The inspected window of T ranged from 5°C to 50°C (in steps of 5°C) and allowed for the reconstruction of the phase diagram. Supplementary Fig. 2 shows the results of this investigation.

The window of nano-star concentrations in which phase coexistence occurs is limited to $c < 117\mu$M: samples at higher concentrations remain homogeneous at all temperatures, not showing any sign of phase separation. The sample at 100 μM and below, instead, exhibit evidence of a re-entrant phase separation process, signalled by the behaviour of the meniscus which appears very well-defined at intermediate T (e.g. between 15 and 40°C for the sample at 78 μM) and progressively disappears on approaching low and high T(e.g. below 15°C and above 40°C for the sample at 78 μM).

Supplementary discussion: Correlation function fit
A convenient quantification of the two-step relaxation can be provided by a non-linear fit of the field autocorrelation function $g_1(t)$ to the functional form

$$g_1(t) = (1-f)e^{-(t/\tau_f)^{\beta_f}} + fe^{-(t/\tau_s)^{\beta_s}} \quad \text{(Supplementary Equation 1)}$$

i.e. to the sum of two stretched exponentials. As an example of the fit quality, Supplementary Fig. 3 shows the fit to the same data reported in the main text at $c = 213\mu$M in the gel-forming region. The parameters f, τ_f, τ_s, β_f and β_s provide a complete characterisation of the dynamic behaviour of the system. The parameter f provides a measure of the amount of correlation which remains in the sample after the fast relaxation process is completed. This amount of correlation relaxes only on the time scale of the slow relaxation process. The times τ_f and τ_s quantify the timescale of the fast and slow processes respectively. The exponents β_f and β_s quantify their stretching amplitude. β_f is always very close to one, while β_s ranges from 0.4 - 0.7. To incorporate the effect of β_s in the evaluation of the typical times of the system we have calculated the average characteristic time via

$$\bar{\tau_S} = \frac{\int_0^\infty te^{-(\frac{t}{\tau_s})^{\beta_s}}dt}{\int_0^\infty e^{-(\frac{t}{\tau_s})^{\beta_s}}dt} = \frac{\tau_s}{\beta_s}\Gamma\left[\frac{1}{\beta_s}\right], \quad \text{(Supplementary Equation 2)}$$

where Γ is the gamma function.

Supplementary discussion: Dynamic Light scattering
Additional samples were investigated by Dynamic Light scattering (DLS) both at low concentrations (for T above and below the coexistence region) and in the homogeneous solution phase. Results, here reported, provide a more complete characterisation of the dynamics.

Low concentration

Measurements of two samples at $c_1 = 39$ μM and $c_2 = 78$ μM were performed (by following the same protocol described in “Materials and Methods”) at low enough Ts, where the samples are homogenous (in agreement with the experimental phase diagram (see Fig. 3a, main text)).

The low-T behaviour of both samples confirms the trend observed at $c = 213$ μM: upon cooling, the decay times of the correlation functions are shifted towards lower values (Supplementary Fig. 4). The correlation functions at these low concentration do not exhibit a clear plateau in the explored T-window providing evidence of the absence of a network of AA bonds. It is also worth noting that the sample at c_2 exhibits a logarithmic decay in an intermediate time interval, possibly suggesting the proximity of the sample percolation threshold.

Homogeneous solution phase

Supplementary Fig. 5 shows the DLS correlation functions for a sample with $c = 117$ μM, i.e. in the homogeneous solution phase, in the region of gel formation (Supplementary Fig. 5(a)) and in the region of gel disruption (Supplementary Fig. 5(b)). As for the $c = 213$ μM case (Fig. 4, main text), the experimental results in the homogeneous gel phase show a progressive slowing down of the dynamics followed, at lower T, by the inverse trend.

Thermal reversibility

Supplementary Fig. 6 contrasts the correlation functions measured during a cooling and the following heating scan to provide evidence that the material is thermally reversible, as expected on the basis of the physical nature of the inter particle bonding.

Supplementary discussion: Effects of Ethidium Bromide
To evaluate how much the Ethidium Bromide (EtBr) used to mark the DNA could shift the phase boundary in T we performed UV absorption experiments to quantify the change in the nano-star self-assembly temperature induced by the presence of EtBr. To measure the melting curve of the DNA strands we used a commercial dual-beam spectrophotometer JASCO v-570 UV/VIS/NIR, endowed with a Peltier thermostat (sensibility = 0.1^oC) which guarantees a

uniform temperature control inside the spectrophotometric cell. Its resolution is of 0.1 nm in the UV-VIS region and 0.5 nm in the NIR region. DNA solutions of nano-stars were prepared at a very low concentrations (0,075 mg/ml) in order to operate in the range in which the relation between absorbance A and concentration is linear, i.e. in which the Beer-Lambert law is valid (for $0.1 < A < 0.8$). For the same reason, we used a thin quartz cell (optical path length: 1 mm). In all absorption experiments, competitor strands were not added to DNA nano-star solutions since the specific aim of these control measurements was only to quantify the effect of the dye on the nano-star melting T.

Two solutions of identically prepared DNA nano-stars were investigated: one was measured as prepared while the other one was marked beforehand with EtBr (0.00129 mg/ml), to provide a comparable EtBr-NS ratio. To properly monitor the melting process, samples were annealed directly in the spectrophotometric cell. The dual-beam operating system of the spectrophotometer allowed for the subtraction of the background. Two reference samples were used for this purpose: a 50 mM NaCl solution in the first case and a solution of 50mM Nacl and EtBr (0.00125 mg/ml) in the second case .

For both samples, we firstly equilibrated the DNA solution at 90°C for 20 minutes with the specific aim of thermally denaturing any possible mismatched double-stranded DNA randomly formed before the annealing, obtaining a homogeneous solution of single-stranded DNA.

Supplementary Fig. 7 shows the comparison between the absorption spectra of nano-stars acquired at 90°C (after a waiting time of 20 min) and at 10°C (for both samples). All curves exhibit the expected absorption peak at 260 nm. A marked hyperchromism of around 20%, resulting from the comparative analysis of the peaks at 90°C (ss-DNA) and 10°C (ds-DNA), allows for the investigation of the melting process by monitoring the absorbance at 260 nm as a function of T.

Following the annealing at 90°C for 20 minutes, we slowly lowered the T down to 10°C, with a cooling rate of 0.67^{o}C/min. Supplementary Fig. 8 shows a comparison of the normalised cooling profiles (in the 90°C - 10°C temperature range) of the solutions of nano-stars in presence and in absence of EtBr. Melting temperatures were estimated from these curves as the temperatures at which the first derivatives of the melting curves exhibit a maximum. The presence of EtBr affects the nano-star melting temperature of DNA, by shifting it towards higher values (of approximately 2°C). We then conclude that the phase boundary shifts at most by a few degrees with the addition of the dye.
